# Association of *MTUS1* with cisplatin response in head and neck squamous cell carcinoma: a retrospective cohort analysis of The Cancer Genome Atlas data

**DOI:** 10.12701/jyms.2026.43.35

**Published:** 2026-05-20

**Authors:** Eun-Kyong Kim, Su Young Oh, So-Young Choi, Tae-Lyn Kim, Heon-Jin Lee, Soyoung Kwak, Su-Hyung Hong

**Affiliations:** 1 Department of Preventive Dentistry, School of Dentistry, Kyungpook National University, Daegu, Korea; 2 Department of Microbiology and Immunology, School of Dentistry, Kyungpook National University, Daegu, Korea; 3 Department of Oral and Maxillofacial Surgery, School of Dentistry, Kyungpook National University, Daegu, Korea

**Keywords:** Drug resistance, *MTUS1*, Squamous cell carcinoma of head and neck, Survival analysis

## Abstract

**Background:**

Cisplatin-based chemotherapy is a mainstay treatment for head and neck squamous cell carcinoma (HNSC); however, resistance to cisplatin contributes substantially to poor clinical outcomes. Identifying biomarkers associated with cisplatin response may improve prognostic assessment and treatment selection.

**Methods:**

We retrospectively analyzed The Cancer Genome Atlas (TCGA)-HNSC dataset to evaluate the association between microtubule associated scaffold protein 1 (*MTUS1*) expression and clinical outcomes, with particular emphasis on patients who were cisplatin-treated. Survival analysis was performed using the Kaplan-Meier curves, and differential expression analysis was conducted separately by comparing patients in disease-specific survival (DSS)-living and DSS-deceased groups. *MTUS1* messenger RNA and protein levels were examined in cisplatin-sensitive oral cancer cell lines and their paired cisplatin-resistant counterparts using quantitative reverse transcription polymerase chain reaction and western blotting. Functional relevance was assessed by small interfering RNA-mediated *MTUS1* knockdown in primary oral squamous cell carcinoma organoids.

**Results:**

MTUS1 protein expression was significantly lower in HNSC tumors than in non-tumor tissues. In the overall TCGA-HNSC cohort, *MTUS1* expression was not significantly associated with survival. However, in patients who were cisplatin-treated, higher *MTUS1* expression was significantly associated with more favorable DSS. *MTUS1* expression was consistently lower in cisplatin-resistant oral cancer cell lines than in their paired cisplatin-sensitive counterparts. Functional experiments further suggested that reduced *MTUS1* expression is associated with decreased cisplatin sensitivity and a resistant phenotype.

**Conclusion:**

*MTUS1* expression may be associated with clinical outcomes in patients with cisplatin-treated HNSC and is related to cisplatin responsiveness. These findings suggest a role for *MTUS1* as a candidate treatment-relevant biomarker and highlight the value of integrating public omics data with experimental validation.

## Introduction

Head and neck squamous cell carcinoma (HNSC) is a common malignancy worldwide and a major cause of cancer-related morbidity and mortality [1,2]. HNSC arises from the mucosal epithelium of the oral cavity, pharynx, and larynx, and its development is associated with multiple risk factors, including tobacco use, alcohol consumption, viral infections, and recurrent genetic alterations in key oncogenic and tumor-suppressive pathways [2,3]. Cisplatin-based chemotherapy remains a cornerstone of treatment for patients with locally advanced HNSC, either as part of concurrent chemoradiotherapy, induction therapy, or in combination treatment strategies [4].

Cisplatin exerts its antitumor effects primarily through DNA damage, ultimately leading to cell cycle arrest and apoptosis [5,6]. However, despite its broad clinical use, treatment outcomes remain limited by intrinsic or acquired cisplatin resistance [7,8]. Multiple mechanisms have been implicated in cisplatin resistance including altered drug transport, enhanced DNA repair, detoxification pathways, dysregulated autophagy, and impaired apoptotic signaling [7-9]. Among these, dysregulation of apoptosis-related pathways is an important contributor to cisplatin responsiveness [7,10]. Although recent genomic studies have identified several candidate genes associated with cisplatin resistance and survival in HNSC and oral squamous cell carcinoma (OSCC), clinically relevant biomarkers linked to patient outcomes and functional cisplatin responses remain insufficiently defined [11,12].

*MTUS1* (microtubule associated scaffold protein 1), also known as *ATIP1* (angiotensin II AT2 receptor-interacting protein 1), has been proposed as a tumor suppressor in human cancers and is located on chromosome 8p21-22, a region that is frequently deleted in multiple malignancies [13-15]. In HNSC, loss of *MTUS1* expression has been associated with unfavorable clinical outcomes [16], and recent studies have demonstrated that *MTUS1* localizes to the outer mitochondrial membrane and exerts antitumor effects by regulating mitochondrial function and reactive oxygen species (ROS)-mediated cell death in HNSC models [17]. Consistent with these findings, *MTUS1* expression is reportedly lower in oral tongue squamous cell carcinoma than in adjacent normal tissues [14]. However, the clinical significance of *MTUS1* in cisplatin-treated HNSC and its functional relationship with cisplatin responses have not yet been clearly established.

Large-scale public cancer datasets provide an opportunity to address this question from both clinical and molecular perspectives. The Cancer Genome Atlas (TCGA) enables integrative analysis of gene expression profiles and clinical outcomes, including survival endpoints, whereas proteomic resources, such as the Clinical Proteomic Tumor Analysis Consortium (CPTAC) allow validation at the protein level [18-21]. These complementary datasets may help determine whether *MTUS1* is associated with general tumor biology, cisplatin-related prognosis, or both. In the present study, we investigated the clinical and functional relevance of *MTUS1* in HNSC with a particular focus on cisplatin response. First, we examined MTUS1 protein expression in HNSC and non-tumor tissues using CPTAC proteomic data. We then analyzed the association between *MTUS1* messenger RNA (mRNA) expression and disease-specific survival (DSS) in the overall TCGA-HNSC cohort and a cisplatin-treated subgroup. Finally, using cisplatin-sensitive oral cancer cell lines and their paired cisplatin-resistant counterparts together with OSCC organoid models, we assessed whether *MTUS1* downregulation is associated with cisplatin resistance. Through this integrated approach, we aimed to clarify the clinical and functional relevance of *MTUS1* in cisplatin response in patients with HNSC.

## Methods

**Ethics statement:** This study was approved by the Institutional Review Board (IRB) of Kyungpook National University Hospital (IRB No: 2021-03-002-001), and written informed consent was obtained from all patients prior to the collection and use of human tissue specimens, in accordance with the principles of the Declaration of Helsinki.

### 1. Analysis of microtubule associated scaffold protein 1 protein expression using the Clinical Proteomic Tumor Analysis Consortium data

MTUS1 protein expression levels in HNSC were analyzed using mass spectrometry-based proteomic data from the CPTAC. The CPTAC HNSC cohort included 179 samples, comprising 70 non-tumor and 109 HNSC tumor tissues. Normalized relative protein expression (nRPX) values for *MTUS1* (UniProt ID: Q9ULD2) were obtained from the CPTAC Data Portal. Samples with missing values were excluded from the analysis. Data normality was assessed using the Shapiro-Wilk test, and because the data were not normally distributed, differences between non-tumor and tumor tissues were evaluated using the Mann-Whitney U test.

### 2. Survival analysis and differential gene expression in The Cancer Genome Atlas-head and neck squamous cell carcinoma cohort based on *MTUS1* expression

Clinical and transcriptomic data were obtained from TCGA-HNSC through the Genomic Data Commons Data Portal. The overall cohort (n=514) and a cisplatin-treated subgroup (n=92) were defined, and transcriptomic expression data were retrieved for each cohort separately. *MTUS1* mRNA expression levels were determined using the Illumina HiSeq RNASeqV2 platform and processed using the RSEM (RNA-seq by expectation-maximization) pipeline. The expression values were log2-transformed to log2(RSEM+1).

For survival analysis, the patients were stratified into *MTUS1*-high and *MTUS1*-low groups using the cohort-specific median *MTUS1* expression value as the cutoff. DSS was evaluated using the Kaplan-Meier curves and compared using log-rank tests. Survival analysis was performed in the overall TCGA-HNSC cohort (n=514) and cisplatin-treated subgroup (n=92).

Differentially expressed gene (DEG) analyses were performed to identify candidate genes associated with clinical outcomes by comparing DSS-living and DSS-deceased patients in each cohort. DEG analysis was conducted using the Welch *t*-test on log2-transformed expression values, with multiple-testing correction by the Benjamini-Hochberg false discovery rate (FDR); adjusted *p*-values are reported as *q*-values. The same DEG pipeline was applied independently to the overall TCGA-HNSC cohort (DSS-living, n=386; DSS-deceased, n=128) and the cisplatin-treated subgroup (DSS-living, n=67; DSS-deceased, n=25).

### 3. Chemicals and reagents

For cell culture, Dulbecco’s Modified Eagle’s Medium (DMEM), fetal bovine serum (FBS), and penicillin-streptomycin were purchased from Invitrogen (Carlsbad, CA, USA). MTT {3-[4, 5-dimethyl-2-thiazolyl]-2, 5-diphenyl-2H-tetrazolium bromide} was obtained from Sigma-Aldrich (St. Louis, MO, USA). QIAzol reagent was purchased from Qiagen (Hilden, Germany), and PCR Master Mix was obtained from Takara Bio (Otsu, Japan). Rabbit anti-*MTUS1* antibody was purchased from Proteintech (catalog #13436-1-AP; Rosemont, IL, USA), and anti-rabbit secondary antibody was purchased from Cell Signaling Technology (catalog #7076; Danvers, MA, USA). Mouse anti-β-actin (horseradish peroxidase [HRP]-conjugated) antibody was obtained from Santa Cruz Biotechnology (Santa Cruz, CA, USA). Cisplatin was purchased from Sigma-Aldrich. A pool of *MTUS1*-specific small interfering RNAs (siRNAs), consisting of two to three target oligonucleotides, was obtained from Santa Cruz Biotechnology.

### 4. Cell lines and organoid cultures

UMSCC1 cells derived from floor of mouth carcinoma were purchased from Merck KGaA (Darmstadt, Germany). The cisplatin-resistant subline UM-CIS was generated from UMSCC1 cells by long-term exposure to cisplatin, as previously described [12]. YD-8 and YD-9 cells originating from tongue and buccal mucosal tumors, respectively, were obtained from the Korean Cell Line Bank (Seoul, Korea). The cisplatin-resistant derivatives, YD-8/CIS and YD-9/CIS, were kindly provided by Professor Jong In Yook (Department of Oral Pathology, Yonsei University College of Dentistry, Seoul, Korea). All cell lines were tested for mycoplasma contamination at 2-month intervals using a CellSafe Mycoplasma PCR Detection Kit (CellSafe Co., Yongin, Korea). The cells were cultured in DMEM containing 10% FBS and 1% penicillin/streptomycin in a humidified atmosphere of 5% CO_2_ at 37°C.

Patient-derived OSCC organoids were established from surgical tumor specimens based on previously reported methods [12,22]. Briefly, tissue specimens were rinsed in ice-cold Advanced DMEM/F12 supplemented with 1×GlutaMAX (35050-061; Gibco, Grand Island, NY, USA), penicillin-streptomycin, 10 mM 4-(2-hydroxyethyl)-1-piperazineethanesulfonic acid, and 100 μg/mL Primocin (ant-pm-1; InvivoGen, San Diego, CA, USA). After mechanical dissociation into approximately 1 to 3 mm³ pieces, the tissues were enzymatically digested with TrypLE (Thermo Fisher Scientific, Waltham, MA, USA) for less than 1 hour. The digested material was centrifuged at 200× *g* for 5 minutes at 4°C, resuspended in complete Advanced DMEM/F12, and passed through a 100-μm cell strainer. Following an additional centrifugation step, the cell pellet was mixed with cold basement membrane extract and seeded as 10-μL droplets onto culture plates. The plates were inverted and incubated at 37°C for 30 minutes to allow matrix solidification, after which prewarmed organoid medium was added. The culture medium was replaced every 2 to 3 days, and the organoids were passaged at 1- to 2-week intervals. Cisplatin responses were monitored for 5 days using a Nikon ECLIPSE Ti microscope (Nikon Imaging Japan Inc., Tokyo, Japan).

### 5. Analysis of messenger RNA and protein expression in oral squamous cell carcinoma cells

*MTUS1* mRNA expression was analyzed using quantitative reverse transcription polymerase chain reaction (RT-qPCR). Total RNA was extracted using QIAzol reagent, and complementary DNA was synthesized using standard reverse transcription protocols. RT-qPCR was performed in three independent experiments, with each condition tested in triplicate, using an ABI 7600 Real-Time PCR System (Applied Biosystems, Foster City, CA, USA). *MTUS1* mRNA expression levels were normalized to those of glyceraldehyde 3-phosphate dehydrogenase (*GAPDH*), and relative expression was calculated using the 2^–ΔΔCt^ method. The primer sequences were as follows: *MTUS1*, forward 5′-CAGGCTGTTCTGCAAGAGTC-3′ and reverse 5′-TCCACAGAAGCTCCTCGTTT-3′; *GAPDH*, forward 5′-AGATCATCAGCAATGCCTCCTG-3′ and reverse 5′-CTGGGCAGGGCTTATTCCTTTTCT-3′.

For MTUS1 protein analysis, total cellular protein was extracted and quantified. Equal amounts of protein (30 μg) were separated on 8% sodium dodecyl sulfate-polyacrylamide gels and transferred onto nitrocellulose membranes. Membranes were blocked with 5% skim milk powder for 1 hour at room temperature and then incubated overnight at 4°C with anti-*MTUS1* antibody. β-actin was used as a loading control. After washing, the membranes were incubated with HRP-conjugated secondary antibody at a dilution of 1:5,000 for 1 hour at room temperature. Protein bands were visualized using an enhanced chemiluminescence detection system. Relative MTUS1 protein levels were assessed by densitometric analysis after normalization to β-actin protein levels.

### 6. Evaluation of cisplatin sensitivity after *MTUS1* small interfering RNA pretreatment in oral squamous cell carcinoma organoids

Cisplatin sensitivity was evaluated in two organoid models derived from different primary OSCC tissues. Approximately 1 month after culture initiation, when the organoids reached a diameter of approximately 300 μm, approximately 70–80 organoids were seeded into each Matrigel dome (Corning, Corning, NY, USA) in 24-well plates. The organoids were then transfected with *MTUS1*-targeting siRNA using Lipofectamine 3000 (Thermo Fisher Scientific) at a final siRNA concentration of 15 nM. Non-targeting control siRNA was used as a negative control. After 24 hours of siRNA pretreatment, the organoids were exposed to cisplatin or the vehicle control (0.1% [v/v] dimethyl sulfoxide in phosphate-buffered saline). Organoids were monitored for an additional 5 days, and their sizes were measured by phase-contrast microscopy at 5× magnification. For each organoid model, the experiment was independently repeated twice, and in each experiment, at least 20 organoids per condition were tracked for quantitative analysis.

### 7. Statistical analysis

All statistical tests were two-sided, and a *p*-value <0.05 was considered statistically significant unless otherwise specified. For multiple-testing analyses, FDR-adjusted *q*-values were used, as indicated. Statistical analyses were performed using Python (version 3.11), as appropriate for each analysis. Data visualization was performed using Matplotlib. The exact statistical details, including sample size, number of biological replicates, statistical tests, and significance levels, are provided in the corresponding figure legends. Independent biological duplicates or triplicates were used in all *in vitro* experiments. Unpaired two-tailed Student *t*-tests were used for *in vitro* comparisons between two groups.

## Results

### 1. MTUS1 protein expression in head and neck squamous cell carcinoma tissues

**Fig. 1.**
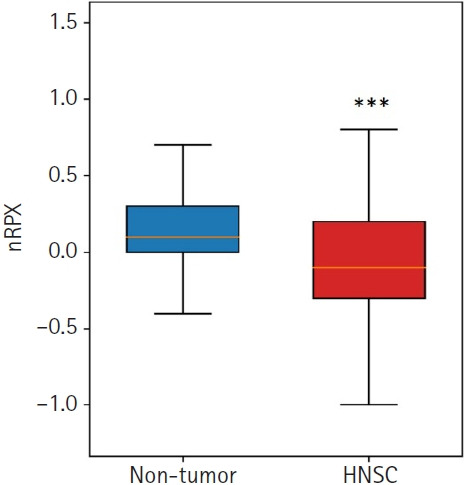
MTUS1 protein expression is reduced in HNSC tissues. Box plots showing MTUS1 protein expression levels in HNSC tumor tissues (n=109) and non-tumor tissues (n=70) from the CPTAC cohort. Protein abundance is presented as normalized relative protein expression (nRPX). The center line indicates the median, the boxes indicate the interquartile ranges (IQRs), and the whiskers extend to 1.5×IQR. Statistical significance is determined using the Mann-Whitney U test (U=5,027, ***p<0.001). MTUS1, microtubule associated scaffold protein 1; HNSC, head and neck squamous cell carcinoma; CPTAC, Clinical Proteomic Tumor Analysis Consortium.

CPTAC proteomic analysis showed that *MTUS1* protein expression was significantly lower in HNSC tumor tissues than in non-tumor tissues, with median nRPX values of −0.100 (interquartile range [IQR], −0.300 to 0.200) and 0.100 (IQR, 0.000–0.300), respectively (Mann-Whitney U test; U=5,027, *p*<0.001; Fig. 1). These findings indicate that MTUS1 was downregulated at the protein level in HNSC tissues.

### 2. *MTUS1* expression patterns in relation to disease-specific survival in The Cancer Genome Atlas head and neck squamous cell carcinoma subsets

**Fig. 2.**
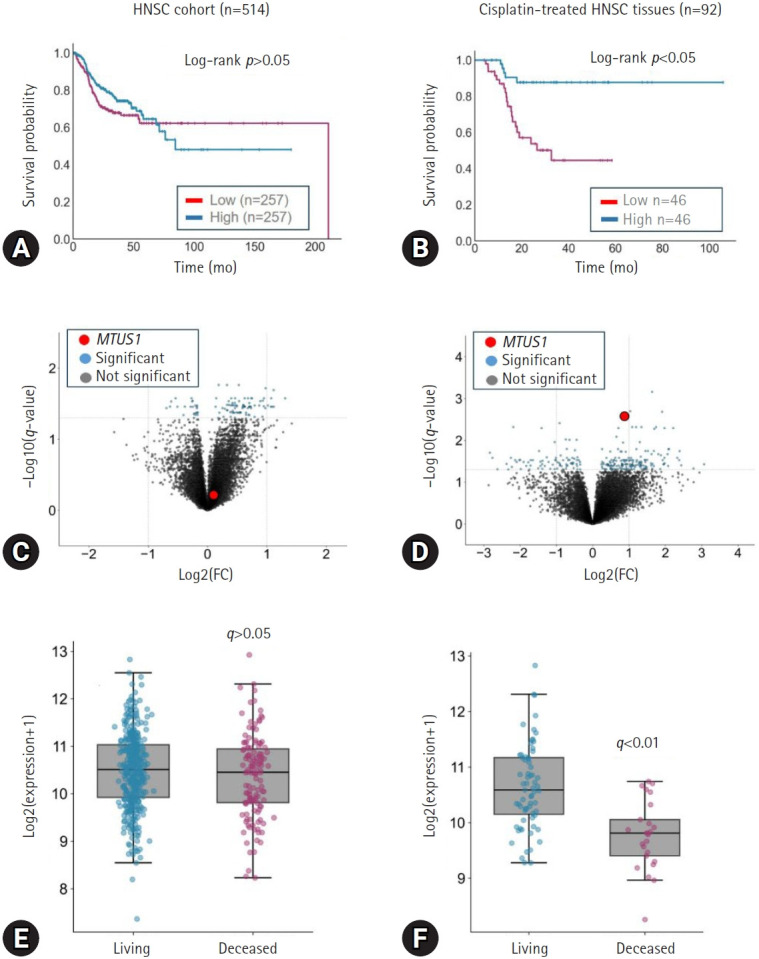
*MTUS1* messenger RNA (mRNA) expression shows a stronger association with disease-specific survival (DSS) in patients from the cisplatin-treated TCGA-HNSC cohort than in those from the overall cohort. (A) Kaplan-Meier curves for DSS in the overall TCGA-HNSC cohort (n=514), stratified into *MTUS1*-high and *MTUS1*-low groups using the cohort-specific median *MTUS1* mRNA expression value as the cutoff (high, n=257; low, n=257). (B) Kaplan-Meier curves for DSS in the cisplatin-treated TCGA-HNSC cohort (n=92), stratified by the median *MTUS1* mRNA expression value (high, n=48; low, n=48). (C) Volcano plot of differentially expressed genes (DEGs) comparing DSS-living and DSS-deceased groups in the overall TCGA-HNSC cohort (overall, n=514; DSS-living, n=386; DSS-deceased, n=128). One hundred and four genes are significantly differentially expressed. (D) Volcano plot of DEGs comparing the DSS-living and DSS-deceased cohort in cisplatin-treated TCGA-HNSC cases (n=92; DSS-living, n=67; DSS-deceased, n=25). Two hundred and forty-four genes are significantly differentially expressed, including 167 genes upregulated and 77 genes downregulated in patients deemed DSS-living. In panels (C) and (D), each dot represents a gene; *MTUS1* is highlighted in red and significant DEGs are shown in blue. Positive log2(FC) values indicate higher expression in patients deemed DSS-living than in those deemed DSS-deceased, and the y-axis shows −log10(*q*-value), where *q*-values are Benjamini-Hochberg FDR-adjusted p-values. (E) Box plot showing *MTUS1* mRNA expression (log2[expression+1]) in patients who are DSS-living and DSS-deceased from the overall TCGA-HNSC cohort (log2(FC)=0.104, *q*>0.05). (F) Box plot showing *MTUS1* mRNA expression (log2[expression+1]) in patients who are DSS-living and DSS-deceased from the cisplatin-treated subgroup (log2(FC)=0.876, *q*<0.005). *MTUS1*, microtubule associated scaffold protein 1; TCGA-HNSC, The Cancer Genome Atlas head and neck squamous cell carcinoma; log2(FC), log2 fold change; FDR, false discovery rate.

To evaluate whether *MTUS1* expression is associated with DSS in HNSC, we analyzed both the overall TCGA-HNSC cohort and a cisplatin-treated subset using parallel survival and transcriptomic approaches. In the overall cohort (n=514), after median-based stratification, there was no significant difference in DSS between the *MTUS1*-high and *MTUS1*-low groups (Fig. 2A). In contrast, among patients who were treated with cisplatin (n=92), the *MTUS1*-high group showed significantly better DSS than the *MTUS1*-low group (Fig. 2B, log-rank *p*<0.05), suggesting that the prognostic relevance of *MTUS1* is more apparent in the context of cisplatin treatment. To further characterize the transcriptome-wide differences associated with DSS outcomes, we performed DEG analysis comparing the DSS-living and DSS-deceased groups. In the overall TCGA-HNSC cohort (overall, n=514; DSS-living, n=386; DSS-deceased, n=128), 104 genes were significantly differentially expressed, whereas *MTUS1* showed only a small effect size and was not statistically significant (log2 fold change [log2FC]=0.104, *q*=0.608) (Fig. 2C, 2E). In the cisplatin-treated subgroup (n=92; DSS-living, n=67; DSS-deceased, n=25), 244 genes were significantly differentially expressed, including 167 upregulated and 77 downregulated genes in patients classified as DSS-living. In this subgroup, *MTUS1* expression was significantly higher in the DSS-living group than in the DSS-deceased group (log2FC=0.876, *q*=0.003) (Fig. 2D, 2F). Together, these findings indicate that the association of *MTUS1* with DSS is limited in the unselected TCGA-HNSC cohort but becomes more pronounced in patients who were treated with cisplatin.

### 3. *MTUS1* is downregulated in cisplatin-resistant oral squamous cell carcinoma cells

**Fig. 3.**
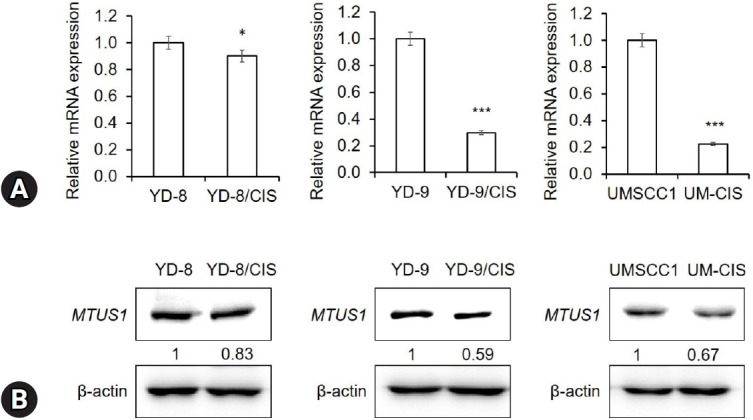
*MTUS1* is downregulated in cisplatin-resistant OSCC cells. (A) Relative *MTUS1* messenger RNA (mRNA) expression in parental and cisplatin-resistant OSCC cell lines, as determined by quantitative reverse transcription polymerase chain reaction. *MTUS1* mRNA levels are normalized to GAPDH mRNA levels and expressed relative to the corresponding parental cells. *p<0.05, ***p<0.001. (B) Representative western blot images showing MTUS1 protein expression in parental and cisplatin-resistant OSCC cell lines. β-actin is used as a loading control. Numbers below the MTUS1 bands indicate relative band intensities normalized to β-actin and expressed relative to the corresponding parental controls. *MTUS1*, microtubule associated scaffold protein 1; OSCC, oral squamous cell carcinoma; *GAPDH*, glyceraldehyde 3-phosphate dehydrogenase.

To determine whether *MTUS1* expression is altered during the acquisition of cisplatin resistance, *MTUS1* mRNA and protein levels were examined in paired parental and cisplatin-resistant OSCC cell lines. RT-qPCR analysis showed that *MTUS1* mRNA expression was significantly lower in cisplatin-resistant cells than in their parental counterparts across all three models, with a modest reduction in YD-8/CIS cells and a more pronounced decrease in YD-9/CIS and UM-CIS cells (Fig. 3A). Western blot analysis revealed a similar pattern, indicating reduced *MTUS1* protein abundance in resistant cells (Fig. 3B). Collectively, these findings indicate that *MTUS1* is downregulated in cisplatin-resistant OSCC cells.

### 4. *MTUS1* knockdown increases cisplatin resistance in oral squamous cell carcinoma organoids

**Fig. 4.**
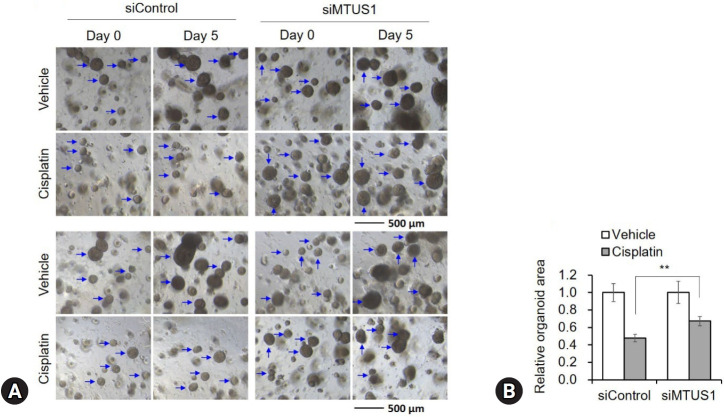
*MTUS1* knockdown increases cisplatin resistance in OSCC organoids. (A) Representative bright-field images of OSCC organoids transfected with control siRNA (siControl) or *MTUS1*-targeting siRNA (siMTUS1) and cultured under vehicle or cisplatin treatment. Images are from day 0 and day 5. Representative images from two independent organoid models are shown. (B) Quantification of relative organoid size in siControl- and siMTUS1-transfected organoids under vehicle or cisplatin treatment. **p<0.01. *MTUS1*, microtubule associated scaffold protein 1; OSCC, oral squamous cell carcinoma; siRNA, small interfering RNA.

To investigate whether the loss of *MTUS1* contributes to cisplatin resistance, *MTUS1* was silenced in OSCC organoids, and the response to cisplatin was assessed. Bright-field images showed that organoids in the siControl and si*MTUS1* groups exhibited similar morphology and growth under vehicle treatment from day 0 to day 5. Upon cisplatin exposure, however, si*MTUS1*-treated organoids appeared to be better preserved than siControl organoids on day 5 (Fig. 4A). Quantitative analysis of relative organoid size showed that under cisplatin treatment, *MTUS1*-knockdown organoids maintained a significantly greater organoid size than siControl organoids (Fig. 4B, *p*<0.01), indicating that cisplatin-induced growth suppression was attenuated by *MTUS1* silencing. These data support the role of *MTUS1* loss in enhancing cisplatin resistance in OSCC organoids.

## Discussion

In this study, we analyzed public proteomic and transcriptomic datasets and experimentally validated them using cisplatin-sensitive and cisplatin-resistant oral cancer models to investigate the clinical and functional relevance of *MTUS1* in HNSC. CPTAC-based proteomic analysis showed significantly lower *MTUS1* protein expression in HNSC tumor tissues than in non-tumor tissues. In parallel, analysis of the overall TCGA-HNSC cohort indicated that the association between *MTUS1* and DSS was limited but became more evident in the cisplatin-treated subgroup. In addition, *MTUS1* expression consistently decreased in cisplatin-resistant OSCC cell lines, and siRNA-mediated *MTUS1* knockdown in OSCC organoids attenuated cisplatin-induced growth suppression. Together, these findings suggest that *MTUS1* downregulation is linked to both HNSC tumor biology and the cisplatin response in a treatment context-dependent manner.

A major strength of this study is the use of publicly available cancer datasets to address biologically and clinically relevant questions that would have been difficult to resolve in a single institutional cohort. TCGA enabled the evaluation of the clinical association of *MTUS1* in both the overall HNSC cohort and a clinically annotated cisplatin-treated subgroup, whereas CPTAC provided protein-level validation in a setting where transcriptome-based tumor-versus-non-tumor comparisons were limited by the relatively small number of non-tumor RNA sequencing (RNA-seq) samples. The complementary use of transcriptomic, proteomic, and clinical annotation data illustrates the practical value of multimodal public data integration for biomarker discovery in translational oncology [19-21].

Our CPTAC analysis showed that *MTUS1* protein abundance was significantly lower in HNSC tumors than in non-tumor tissues, supporting the biological relevance of *MTUS1* loss in HNSC. This interpretation is consistent with recent evidence showing that *MTUS1* localizes to the outer mitochondrial membrane and interacts directly with mitofusin-2 in HNSC cells. Through this mitochondrial axis, *MTUS1* increases ROS generation and disrupts mitochondrial homeostasis, as reflected by reduced mitochondrial membrane potential and respiratory activity. These changes facilitate Bax translocation and cytochrome c release, leading to caspase-3 activation and subsequent pyroptotic cell death [17]. In addition, the epigenetic and posttranscriptional regulation of *MTUS1* has recently been implicated in HNSC/OSCC progression. In HNSC, fat mass and obesity-associated protein was shown to demethylate m6A sites within the 3′ untranslated region of *MTUS1*, thereby reducing *MTUS1* mRNA stability and translational efficiency and ultimately promoting proliferation, migration, and tumor growth [23]. In OSCC, recent evidence further demonstrates that YTH N6-methyladenosine RNA binding protein F2 recognizes m6A-modified *MTUS1* transcripts and accelerates their degradation, leading to the suppression of *MTUS1* expression, mitochondrial dysregulation, and malignant progression [24]. Together, these findings suggest that reduced *MTUS1* expression in HNSC/OSCC arises through coordinated epigenetic and posttranscriptional mechanisms and provides a biologically plausible framework for our observation that *MTUS1* loss is associated with a cisplatin-resistant phenotype. Therefore, our observation of reduced MTUS1 protein expression in CPTAC tumor tissues provides clinically relevant proteomic support for this emerging mechanistic axis, although the direct downstream pathway linking MTUS1 depletion to cisplatin resistance in our experimental models remains unclear.

Notably, the clinical relevance of *MTUS1* became more apparent after restricting the analysis to patients treated with cisplatin. This pattern indicates that *MTUS1* may be better understood as a treatment context-dependent candidate biomarker linked to platinum responsiveness, rather than a broadly applicable survival marker for all HNSCs. This interpretation is also supported by recent transcriptomic evidence indicating that cisplatin resistance in HNSC is more closely associated with molecular programs related to DNA damage responses and cell cycle arrest than with impaired drug uptake itself [8]. More broadly, cisplatin responsiveness is influenced not only by drug uptake but also by DNA damage responses, mitochondrial stress signaling, and downstream cell-death pathways [6,7,10]. In this setting, loss of *MTUS1* may not be relevant as a general prognostic factor but as a marker associated with cellular states that influence cisplatin response under a defined treatment context. Although direct evidence linking *MTUS1* downregulation to cisplatin resistance remains limited, previous studies support the role of *MTUS1* in mitochondrial regulation [17]. Although the downstream mechanism remains to be fully clarified, the concordance between the subgroup-specific clinical signals and functional results from resistant cell lines and organoid knockdown experiments supports the view that *MTUS1* is more likely to be a treatment-relevant candidate biomarker than a purely correlative finding.

This study had several limitations. First, the public data analyses were retrospective and depended on the completeness of treatment and outcome annotations, particularly in the cisplatin-treated subset, which remained modest in size. In addition, because of the nature of public datasets, detailed treatment-related variables, including cisplatin dose, administration schedule, and concurrent therapies, such as combination chemotherapy or radiotherapy, may not have been fully captured. Therefore, the subgroup findings should be interpreted cautiously as hypothesis-generating and require validation in additional well-annotated cohorts. Second, TCGA RNA-seq data were generated from bulk tumor tissues and therefore did not distinguish tumor cell-intrinsic expression from stromal or immune contributions. Third, CPTAC and TCGA capture different molecular layers and partially different case sets; therefore, transcript-protein concordance should not be assumed automatically. Finally, although our experimental models support a functional role of *MTUS1* in cisplatin response, the downstream pathway linking *MTUS1* loss to cisplatin resistance remains to be defined more directly, and additional *in vitro* validation in broader experimental settings is needed to further substantiate the mechanistic relationship.

In conclusion, our data suggest that *MTUS1* serves as a treatment context-dependent candidate biomarker in cisplatin-treated HNSC rather than as a general prognostic marker in all HNSC cases. By integrating CPTAC proteomics, TCGA transcriptomics, and functional validation in cisplatin-resistant cell lines and OSCC organoids, we found that *MTUS1* loss was associated with reduced protein expression in tumors and with a phenotype consistent with diminished cisplatin sensitivity. More broadly, this study illustrates how public cancer datasets can serve not only as discovery tools but also as a framework for generating clinically meaningful, subgroup-oriented hypotheses that can be tested experimentally.
